# Building Roof Segmentation from Aerial Images Using a Line-and Region-Based Watershed Segmentation Technique

**DOI:** 10.3390/s150203172

**Published:** 2015-02-02

**Authors:** Youssef El Merabet, Cyril Meurie, Yassine Ruichek, Abderrahmane Sbihi, Raja Touahni

**Affiliations:** 1 IRTES-SeT, University of Technology of Belfort-Montbeliard, 13 rue Ernest-Thierry Mieg, 90010 Belfort cedex, France; 2 LASTID Laboratory, Département de Physique, Faculté des Sciences, Université Ibn Tofail, B.P 133, 14000 Kénitra, Maroc; 3 Univ Lille Nord de France, F-59000 Lille, IFSTTAR, LEOST, F59650 Villeneuve d'Ascq, France; 4 National School of Applied Sciences of Tangier (ENSAT), Abdemalek Essaadi University, B.P. 1818, 90000 Tangier, Maroc

**Keywords:** orthophotoplan, image segmentation, watershed, region merging, roof segmentation, 2D roof ridge modeling

## Abstract

In this paper, we present a novel strategy for roof segmentation from aerial images (orthophotoplans) based on the cooperation of edge- and region-based segmentation methods. The proposed strategy is composed of three major steps. The first one, called the pre-processing step, consists of simplifying the acquired image with an appropriate couple of invariant and gradient, optimized for the application, in order to limit illumination changes (shadows, brightness, *etc*.) affecting the images. The second step is composed of two main parallel treatments: on the one hand, the simplified image is segmented by watershed regions. Even if the first segmentation of this step provides good results in general, the image is often over-segmented. To alleviate this problem, an efficient region merging strategy adapted to the orthophotoplan particularities, with a 2D modeling of roof ridges technique, is applied. On the other hand, the simplified image is segmented by watershed lines. The third step consists of integrating both watershed segmentation strategies into a single cooperative segmentation scheme in order to achieve satisfactory segmentation results. Tests have been performed on orthophotoplans containing 100 roofs with varying complexity, and the results are evaluated with the VINETcriterion using ground-truth image segmentation. A comparison with five popular segmentation techniques of the literature demonstrates the effectiveness and the reliability of the proposed approach. Indeed, we obtain a good segmentation rate of 96% with the proposed method compared to 87.5% with statistical region merging (SRM), 84% with mean shift, 82% with color structure code (CSC), 80% with efficient graph-based segmentation algorithm (EGBIS) and 71% with JSEG.

## 1. Introduction

Image segmentation is one of the most difficult problems in the field of image analysis and computer vision. It consists of partitioning an image into a set of regions according to certain properties, such as intensity (gray level), texture or color. Several general-purpose algorithms and techniques have been developed for image segmentation in the literature. They can be generally grouped into two main categories: edge-based and region-based segmentation. The first category consists of detecting the dissimilarity and transitions between objects in the image. It generally assumes *a priori* knowledge of a model to detect and operate on very localized edges. Its major drawback concerns the sensitivity to noise, texture or illumination changes [1], which leads to false edges that are generally not closed [2–4]. Thus, these approaches do not lead directly to a good segmentation [2]. The second category of segmentation methods consists of grouping adjacent pixels according to a certain similarity criteria. The advantage of this approach is that it produces closed and connected segments. Among the morphological techniques most frequently used, one can cite split/merge and region growing (in which we find the watershed algorithm used in this paper) [5]. However, the localization of the region boundaries remains generally less accurate [6,7]. This can be justified by the difficulty in defining the best criteria or parameters in the split/merge or pixel aggregation strategies [4,8], generally set on the basis of several tests and experiments on the studied images.

Generally, each segmentation method has its own limitations and advantages in terms of applicability, suitability, performance and computational cost. It is difficult to fulfill simultaneously all of these qualities by a single segmentation algorithm. For specific applications, such as roof detection in aerial images, using a single segmentation technique (edge- or region-based segmentation) is generally insufficient to obtain satisfactory results. The trend towards integrating several techniques seems to be the best way forward. Indeed, a cooperation between different segmentation techniques can be suitable in order to exploit their advantages, reduce the problems that arise in each individual method and, then, improve the quality of the segmentation. Many segmentation approaches combining results generated from several techniques have emerged in recent years. Indeed, the robustness of the treatment is greatly improved by the use of several hybrid segmentation methods [5,9–11]. Segmentation based on region-edge cooperation permits one to exploit the two pieces of complementary information in order to integrate them into the same segmentation scheme and, thus, contribute to a better segmentation [12–15]. An extensive review of cooperative strategies for image segmentation combining region and edge information can be found in [10,13].

Unfortunately, the choice of the segmentation method is closely related to the particularities of the image and the considered application, and many segmentation approaches are not suitable for noisy environments, such as aerial and satellite images. For that, a great variety of methods for remote sensing data segmentation have been developed and can be classified into three groups: (1) image based; (2) LiDAR based; and (3) a combination of both image and LiDAR based [16–19]. In particular, in the field of aerial image segmentation, wherein the proposed segmentation approach will operate, several segmentation techniques have been proposed and usually have been combined in the last few years. One can cite region growing-based approaches, such as watershed transform, the region merging strategy and the split/merge technique. All of these techniques are developed to detect objects of interest in aerial images, such as roads [20,21], forests [22,23] and buildings [24–27].

Ali in [28] proposed an automated methodology for the detection and description of 2D building footprints from a single color aerial orthoimage. This method is based on a mean shift segmentation algorithm and Canny edge detection algorithm. After mean shift segmentation, edge pixels that are detected using the Canny operator and that form a closed boundary are converted into polygons in order to obtain the building shape after some refinement.

The authors in [4] proposed a cooperative approach to multiband satellite image segmentation. The method is based on cooperation between region growing segmentation and edge segmentation. The edge segmentation is performed first to obtain an edge map. Afterwards, this information is used by multispectral region growing segmentation as an additional criterion in decision-making. Integration of edge information consists of selecting the positions of seed pixels and defining the segmentation criterion. Mohamed *et al*. in [29] proposed an unsupervised cooperative approach for satellite image segmentation combining two segmentation methods: self-organizing maps (SOMs) and fuzzy C-means (FCM). The combination of these methods allows one to create an unsupervised, parameter-free approach.

The watershed algorithm is largely employed in the field of aerial and satellite image segmentation. In Chen *et al*. [30], the proposed method uses the watershed algorithm combined with a region merging procedure. First, a homogeneity gradient image is produced. Then, an efficient watershed transform is employed to gain the initial segments. Finally, an improved region merging process is applied to merge the initial segments. For that, a strategy is proposed to minimize the overall heterogeneity increased within segments at each merging step. The authors in [31] proposed a multi-spectral satellite image segmentation based on the watershed algorithm combined with a region merging technique.

Although many studies have been published on aerial and satellite image segmentation, none of them focuses specifically on roof segmentation. Segmenting roofs in different regions of interest (sections of roofs, chimneys, roof light, *etc*.), which is the main aim of this paper, has unfortunately received much less attention. Indeed, roof segmentation plays a crucial role in the field of the automatic generation of 3D buildings, where a large number of studies have been performed in the context of automatic building (roof) extraction. Among recent limited studies in the field of roof segmentation, one can cite the work presented in [32]. The authors proposed a building extraction and segmentation method from high-resolution color imagery using edge flow-driven active contours and the Jsegunsupervised segmentation algorithm [33]. First, this method consists of denoising and using a color quantization by anisotropic diffusion and clustering. Then, building boundaries are extracted by an active contour driven by edge-flow. Finally, building roofs are segmented by the Jseg segmentation algorithm.

In this overall context, the work presented in this paper is developed in a global approach that consists of recognizing roofs extracted from aerial images using a knowledge database and bending out 3D models automatically generated from geographical data. The main step presented in this paper consists of segmenting roof images into different regions of interest in order to provide several measures of roofs (section of roofs, chimneys, roof light, *etc*.). For that, a cooperative segmentation approach that integrates watershed regions and watershed lines is proposed. This technique will combine the advantages of both segmentations obtained by the watershed algorithm to identify and extract from aerial images (orthophotoplans) the regions of interest that are more faithful to real roof objects.

The contribution of this paper is threefold: (1) an automatic method for choosing an appropriate couple of colorimetric invariant/gradient for image simplification purposes is adopted; (2) an efficient region merging strategy based on 2D roof ridge modeling and region features adapted to orthophotoplan particularities is developed; and (3) a cooperative segmentation approach of edge- and region-based segmentation methods is finally proposed.

This work is structured as follows: In Section 2, we present the global overview of the proposed orthophotoplan segmentation approach. Section 3 describes the strategy for choosing the optimal couple of colorimetric invariant/gradient for image simplification purposes. Section 4 presents the watershed region-based and watershed line-based segmentation methods. In this section, the paper starts by presenting the watershed region-based segmentation method, followed by the 2D roof ridges modeling technique, then the region merging strategy and, finally, the watershed line-based segmentation method. In Section 5, we describe the cooperation technique based on the two watershed region- and line-based segmentations. Experimental results and a comparison with popular segmentation methods are shown in Sections 6 and 7, respectively. Section 8 concludes the paper and presents future works.

## 2. Overview of the Proposed Segmentation Approach

**Figure 1.**
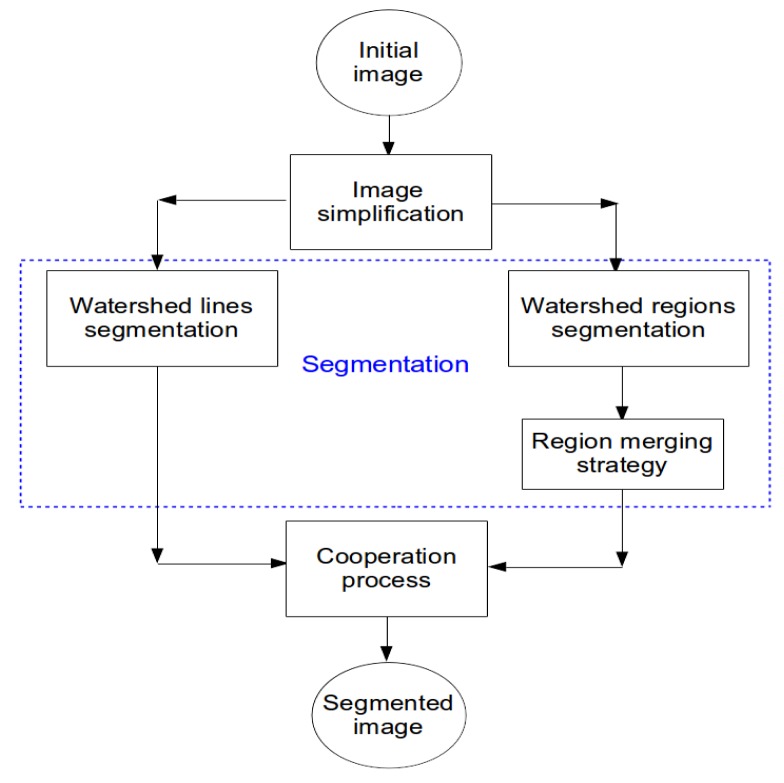
Flowchart of the proposed orthophotoplan segmentation approach.

Generally, cooperative approaches that consist of combining two or more techniques are widely used to get the desired output for particular applications. In such approaches, the complementarity of edge and region information is taken into account to reach a more precise segmentation that is faithful to the desired real objects. Several cooperative approaches have been developed in the state-of-the-art, and three categories can be identified: sequential cooperation, mutual cooperation and results cooperation [4]. These approaches differ by how the regions and edges are integrated and what level of processing is carried out in the integration. In sequential cooperation, the region (or edge) information is extracted firstly and is then used by the edge (or region) -based method to make the segmentation criteria or parameters more robust [34,35]. For mutual cooperation, the two segmentation techniques are carried out in parallel, but with a mutual exchange of information [8,36]. This information exchange assists each of the two segmentation techniques. In the case of results cooperation, both region and edge segmentations are carried out independently. Then, the region map and edge map are fused to achieve meaningful segmentation [37,38]. The orthophotoplan segmentation approach proposed in this paper and described below belongs to this last cooperative approach. It consists of integrating edge-based segmentation and region-based segmentation into the same cooperation process, in which both segmentation techniques are assured by the watershed algorithm (watershed lines and watershed regions). Figure 1 illustrates the main steps of the proposed segmentation method.

- Image simplification: This part is a pre-processing step that consists of simplifying the input image with the optimal couple of colorimetric invariant/color gradient optimized for the application. The use of an appropriate couple of invariant/gradient permits one to limit artifacts and illumination changes (shadows, brightness, *etc*.) affecting the images and, thus, to increase the robustness of roof segmentation.
- Image segmentation: This step includes two parallel and independent image segmentation processes based on the watershed algorithm. On the one hand, the simplified image is partitioned into primitive regions using watershed regions coupled with an efficient region merging strategy. This region merging procedure includes a merging criteria based on 2D modeling of roof ridges and region features adapted to orthophotoplan particularities. The 2D modeling technique consists of defining a 2D model describing the roof ridges by segments for which the extremities are the nodes connecting the roof ridges where each roof ridge is characterized by one and only one segment. On the other hand, the simplified image is segmented using watershed lines in which no post-processing is applied.
- Cooperation process: This step consists of integrating both segmentation results obtained via the watershed algorithm into a single cooperative segmentation scheme to achieve more satisfactory segmentation results. The cooperation strategy belongs to the category of hybrid techniques. Indeed, the proposed region/edge cooperative process is able to incorporate the advantage of each segmentation method and, thus, to achieve reliable segmentation results.

## 3. Image Simplification

In our application, orthophotoplans contain a certain heterogeneity in terms of light, illumination changes, shadows, *etc.*, applying the watershed algorithm directly on the images without any pre-processing step, making the segmentation delicate and difficult to perform. To deal with these drawbacks and therefore to extract the different regions of interest of the roofs correctly, we adopt a common strategy consisting of simplifying the input image with a suitable colorimetric invariant [39–43]. Indeed, in the last few years, color invariance has generated much interest and continues to engage the field of computer vision. For example, one can cite the use of colorimetric invariant for image matching [41], motion estimation in video sequences [42], feature extraction and re-identification of individuals in a transport environment [39], enhancing the monitoring of points of interest in color images [43], building detection from aerial imagery [44], *etc*. In this work, we investigate the effect of colorimetric invariants on the outcome of watershed-based orthophotoplan segmentation. In other words, the objective is to show how using color invariance could limit the artifacts present in the acquired images (noise and unimportant fine-scale details). This color invariance analysis is associated conjointly with the use of different gradient operators, since the gradient image, which is the input of the watershed algorithm, is strongly dependent on the color invariant used. Thus, our analysis consists of determining the optimal couple of color invariant/gradient in the image simplification step.

**Figure 2.**
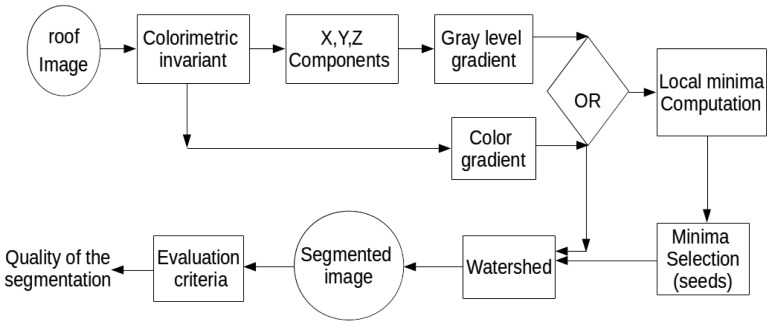
Synopsis of the strategy for choosing the optimal couple of invariant/gradient.

The first step of our orthophotoplan segmentation approach consists of simplifying the input image with an appropriate couple of colorimetric invariant/gradient. Figure 2 illustrates the synopsis of the strategy that permits choosing the optimal couple of colorimetric invariant/gradient applied to the input image of the watershed algorithm. It is composed of several steps: First, we apply a colorimetric invariant on the initial image. After that, we calculate a color gradient or a gray level gradient on the simplified image. In the case of the gray level gradient, we extract the three components of the simplified image and calculate the gradient on these components. The next step consists of using the watershed with the gradient image and a seed image. Finally, we obtain the segmented image and evaluate the quality of the segmentation, with the VINETcriterion, according to a reference segmentations. Tests have validated the interest in using an appropriate couple of colorimetric invariant/gradient in the segmentation process.

**Figure 3.**
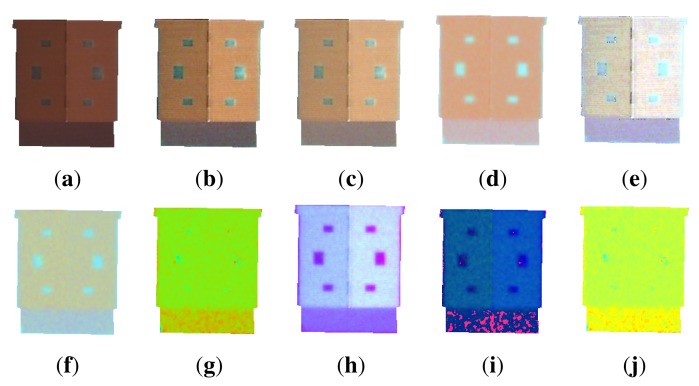
Example of colorimetric invariants. (**a**) Original image; (**b**) with affine normalization; (**c**) with greyworld normalization; (**d**) with c1c2c3; (**e**) with RGB-rang; (**f**) with comprehensive color normalization; (**g**) with l1l2l3; (**h**) with standard L2; (**i**) with hslspace; and (**j**) with A1A2A3.

For this, 24 colorimetric invariants in the literature have been tested: greyworld normalization (called greyworld in Figures 13 and 14) [45], RGB-rang [46], affine normalization (called affine in Figures 13 and 14) [47], intensity normalization (called chromaticity in Figures 13and 14) [46], comprehensive color normalization (called comprehensive in Figures 13 and 14) [45], c1c2c3 [40,41], m1m2m3 [41], l1l2l3 [40,41], l4l5l6 [48], A1A2A3 [43], c4c5c6 [48], HSL, MaxRGB [46], CrCgCb [43], color constant color indexing (called CCCI in Figures 13 and 14) [46], m4m5m6 [43], standard L2 (called L2 in Figures 13 and 14) [43], maximum-intensity normalization (called Mintensity in Figures 13 and 14) [49], reduced coordinates [50], CrCb [40,43], opposite colors (o1o2) [40,43], saturation (S) [41], log-hue [46] and hue (H) [41,50]. Figure 3 illustrates the visual difference of some colorimetric invariants applied on the initial image.

**Figure 4.**
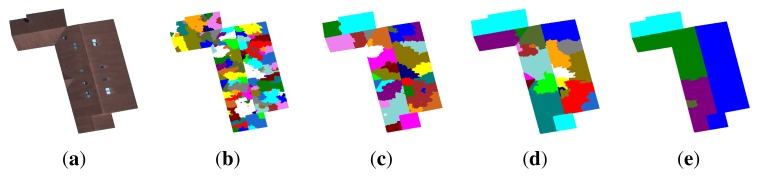
Example of seed reduction. (**a**) Initial image; segmented images with (**b**) all local minima (136 regions); (**c**) *α* = 1, *β* = 5 (38 regions); (**d**) *α* = 10, *β* = 15 (21 regions) and (**e**) *α* = 1, *β* = 50 (under-segmentation with only seven regions).

**Figure 5.**
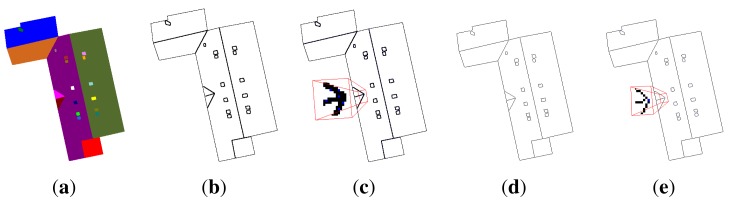
Edge extraction: the influence of the thinning process. (**a**) Segmented image of Figure 4a; (**b**) extracted edges without thinning; (**c**) the corresponding 2D model (edges characterized by 329 segments); (**d**) extracted edges with thinning; and (**e**) the corresponding 2D model (edges characterized by only 83 segments).

To partition the image into homogeneous regions, the watershed uses the gradient image that we propose to calculate the simplified image with a colorimetric invariant. Thus, the segmentation result is significantly influenced by the colorimetric invariant and, then, by the gradient calculation. In order to study the influence of the gradient image on the segmentation results obtained by the watershed algorithm, and, thus, to define the best couple of invariant/gradient for our application, several gradient-calculation techniques are tested and evaluated. They can be grouped into two categories: gray level gradient and color gradient. The grey level gradient is computed using different local edge detection techniques that are primarily based on applying edge-detection operators, which are distinguished by the filter used. Six simple operators are considered: (1) the first derivative of the image (denoted GradientF in Figure 14); (2) the morphological gradient corresponding to the subtraction between the dilated image and the eroded image (denoted GradientM in Figure 14); (3) NonMaximaSuppression (the non-maxima values from the magnitude of the gradient); (4) the Sobel; (5) Roberts and (6) Prewitt operators; and two more complex operators are used: (7) Deriche; (8) Shen. For color gradients, they can be classified into three main categories: (i) fusion methods or marginal methods; (ii) perceptual methods; and (iii) gradient tensor or gradient vector methods. For the first category, we have used: (1) the marginal gradient (denoted GradientCin Figure 13) [51]; and (2) the morphological gradient corresponding to the subtraction between dilation and erosion using a lexicographical order (denoted GradientMCin Figure 13). For the second category, we have tested three color gradients: (3) Sobel color calculated on the color image (denoted SobelC in Figure 13); (4) Sobel color calculated in TLScolor space (denoted SobelTLS in Figure 13); (5) Carron [52]. For the last category, we have used (6) Di Zenzo's gradient operator [53].

## 4. Watershed Segmentation

Among all existing segmentation approaches, we have chosen a mathematical morphology framework. Indeed, region growing-based segmentation methods seem to be more adapted when considering the objectives of our application. In the following, we propose a segmentation method based on the watershed algorithm, which generally provides satisfactory results compared to other implemented segmentation techniques. In fact, the watershed algorithm has several advantages: (1) the proper handling of gaps; (2) the placement of boundaries at the most significant edges; and (3) the produced regions are closed and connected (whereas edge-based techniques usually lead to disconnected boundaries that need post-processing to produce closed regions). Although the watershed is usually considered as a region-based approach, De Smet *et al*. [54] pointed out that watershed transformation has proven to be a powerful basic segmentation tool that can hold the attributed properties of both edge detection and region growing techniques. Many sequential algorithms have been developed to compute watershed transforms [55–57]. They can be divided into two classes: the first one is based on the specification of a recursive algorithm proposed by Vincent and Soille [57], and the other one is based on distance functions (topographical distance) introduced by Meyer [55]. For more details, an extensive review of watershed algorithms can be found in [56]. In this paper, both Meyer's and Vincent–Soille's algorithms are used and integrated into the proposed single cooperative segmentation scheme. The final segmentation calculated by the proposed orthophotoplan segmentation approach is mainly based on the segmentation results produced by Meyer's algorithm. Vincent–Soille's algorithm is used in order to deal with one drawback of the first one.

### 4.1. Segmentation Using Watershed Regions

In order to obtain a preliminary orthophotoplan segmentation, we have used Meyer's algorithm [55]. This watershed segmentation technique is based on a simple heuristic that consists of analyzing the gray level of the image pixels in ascending order. Performing region growing, the watershed region technique uses a gradient image, calculated on the simplified image, and a seed image, calculated from the gradient image. The growing process determines the region associated with each seed, by gathering into the region the pixels that are the closest to the corresponding seed, provided that a certain homogeneity in the gray level is satisfied. Even if the watershed region-based segmentation results, using the appropriate couple of invariant/gradient optimized for the application, are interesting, the images are over-segmented. To overcome this shortcoming, many helpful tools have been proposed in the literature. Most of them consist of: (1) filtering the input image; and/or (2) selecting only a reduced and significant set of local minima; and/or (3) merging all non-significant regions of the obtained segmented image. We point out that all of these solutions have been taken into consideration in our orthophotoplan segmentation approach. In fact, the initial image is simplified by applying a suitable colorimetric invariant. Regarding the selection of seeds, several efficient algorithms have been proposed in the literature to reduce the number of irrelevant local minima. One can cite the simplest one that corresponds to an interactive selection by the user [58] or by using *a priori* knowledge of the image [59]. The dynamic approach proposed in [60] consists of ordering all local minima and selecting only those above a threshold. In [61], this dynamic approach consists of providing an intuitive selection scheme controlled by a single parameter τ using grayscale reconstruction.

In this paper, we have opted to use the method proposed in previous works [62] and which offers generally good results. The basic idea of the selection of the local minima is based on two parameters *α* and *β,* that define the percentage of seeds given by $\frac{\alpha }{\beta }\times 100$. Reducing the number of local minima (*i.e.,* using an optimal couple of (*α, β*)) considerably reduces the over-segmentation of the image. Figure 4 illustrates some segmentation results with different percentages of seeds. Note that the couple (*α* = 10, *β* = 15) appears to be a good compromise between sufficient attenuation of over-segmentation and proper restitution of the main structures of the roof. Indeed, the corresponding segmentation is pertinent, because all roof sections are present with a low over-segmentation (21 regions). In order to improve the quality of the segmentation results more and, thus, to limit the problem of over-segmentation further, keeping all of the regions of interest, we merge all non-significant regions. Before describing the proposed region merging process, we detail a 2D roof ridges modeling technique for which we need to calculate one of the merging criteria.

#### 4.1.1. 2D Modeling of Roof Ridges

The basic idea of the 2D roof ridges modeling process consists of defining a 2D model describing roof ridges by segments, where each ridge is characterized by one and only one segment. The extremities of the segments correspond to the nodes connecting the roof ridges. The modeling process depends exclusively on the segmentation results obtained in the previous step. It starts by extracting the edges of the pre-segmented image. After that, it transforms all of the edges into segments where the extremities are the nodes that connect the roof ridges. More precisely, the modeling strategy is composed of four steps described as follows: (1) extract the edges of the pre-segmented image; (2) extract all of the segments and the intersection points characterizing each roof; (3) subdivide the segments that do not characterize the real shape of a roof by modifying/optimizing the position of the intersection points; (4) add tolerance points. These four steps are detailed below.

##### Edge Extraction in the Pre-Segmented Image

The first step in the modeling process concern edge extraction in the pre-segmented image. It is performed by scanning the image in the two horizontal and vertical directions. A pixel located in a transition state (between two different regions) is considered an edge pixel. This allows one to extract edges that are multi-pixel-wide. In order to obtain a good quality 2D model, the edge width must be reduced to one pixel. For this purpose, we use a skeletonization thinning algorithm introduced in [63]. The thinning process consists of applying a recursive algorithm using a morphological concept based on the application of successive thinning until obtaining a stable structure that is not able to be thinned (*i.e*., lines with single pixel thickness). Figure 5 illustrates an example of 2D models obtained without/with thinning. One can see that the thinning process permits one to decrease the number of segments characterizing a roof in the 2D model, so that each roof ridge is represented by a unique segment. Indeed, in Figure 5d, the 2D model characterizing the roof is composed of 83 segments corresponding to the presence of 83 roof ridges. On the contrary, if the thinning process is not used, some roof ridges are characterized by several segments, and hence, the 2D model does not fit the real shape of the roof. This situation is illustrated in Figure 5b where the same roof is characterize by 329 segments.

##### Extraction of Segments

**Figure 6.**
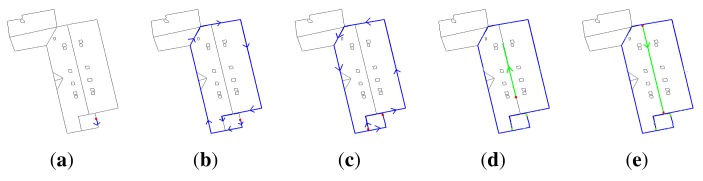
Illustration of the creation of segments. (**a**) The choice of an arbitrary starting point; (**b**) the addition of the best adjacent points; (**c**) the path direction that is reversed; (**d**) the choice of another arbitrary starting point and the addition of the best adjacent points (green segment); and (**e**) the path direction that is reversed (green segment).

This step consists of exploiting edges previously extracted in order to transform them into segments. Firstly, the procedure begins by choosing, arbitrarily, an edge point as a starting point. Then, we add successively to this point all neighbor points until a “closed” segment is obtained. In other words, the creation of a segment is stopped once the adding point procedure tries to add a point that coincides with the starting point or a point that has already been referenced in an existing segment. The difficulty is how to choose the best point to add and then the best path direction, when the last added point connects more than two ridges. To resolve this situation, we consider the current direction of the segment. Thus, the best point *P* to add to the last added point N is chosen by maximizing the scalar product between the vector formed by the points *N* and *P* and the vector formed by the last five points added to the segment (the current direction of the segment). Algorithm 1 describes the procedure for choosing the best point to add when the last added point is a node connecting more than two ridges. Once no more adjacent non-affected points appear, the path direction is reversed from the last added point in order to adjust the starting point that was chosen arbitrarily. A point on which the path direction is reversed or a creation of a segment is stopped is considered an intersection point. These tow points correspond to the extremities of the segment. The process is repeated until there are no points to affect. Figure 6 illustrates an example of the creation of segments.


**Algorithm 1:** Choice of the best point to add to a segment
**begin** vectorLength(point A; point B) ← length of the vector connecting the point A to the point B seg ← the current segment pt ← the last point added to the segment seg ListPoint ← adjacent points to the point pt PtF ← last point of the segment seg ptD ← (size(seg) > 5) ? 5th point before ptF: beginning of the segment seg maxValue ← 0 **if**
*((ptD AND ptF) not NULL)*
**then**  **for**
*each point pts of ListPoint*
**do**   denominator ← vectorLength(pt ; pts)*vectorLength(ptD ; ptF)   tempValue ← scalar product [pt ; pts] with [ptD ; ptF] / denominator   **if**
*(tempValue* > *maxValue)* then    maxValue ← tempValue    BestPoint ← pts


##### Segment Subdivision

**Figure 7.**
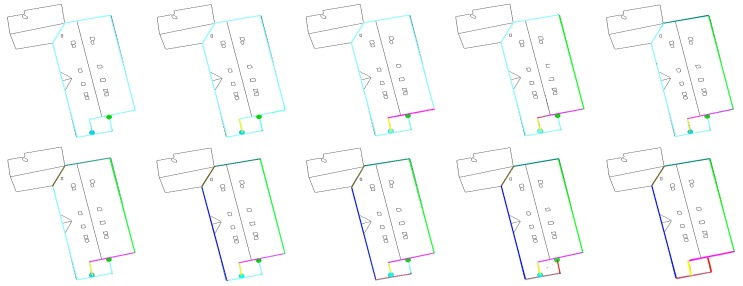
Illustration of segment subdivision when the segment represents several ridges.

The 2D model defined by segments provided by the previous steps often presents an undesirable situation in which several ridges are characterized by a single segment, and hence, it does not correctly represent the real shape of the corresponding roof. This is due to the stop conditions of the segment creating process. This drawback is illustrated in the first image of Figure 7, where all of the ridges of the outer part of the roof are represented by a single segment, resulting from the segment creation procedure (the aqua segment in Figure 6d). To deal with this drawback, each segment that represents several ridges is subdivided into a set of segments in which each one represents only one ridge (the last image in Figure 7). This is performed by scanning, point by point (from one extremity to the other), the segment in order to find intersection points. An intersection point is a common point for two ridges or more. Given a segment [*startPt, endPt*] to be subdivided, the procedure starts by determining the first intersection point *pt* that maximizes the length sum of the vectors formed respectively by the points *startPt* and *pt* and the points *pt* and *endPt* (Algorithm 2). Then, the new segments [*startPt, pt*] and [*pt, endPt*] are processed in the same way. The procedure is repeated, until all intersection points are identified. At the end of the procedure, the desired segments are obtained by the computed intersection points, where two successive intersection points define a segment. Figure 7 illustrates the subdivision process, where the final segments are marked with different colors. It is important to note that when a point *pt* maximizes the length sum for a segment, we apply an additional test with the threshold before to identify it as an intersection point (Algorithm 2). This test allows avoiding the creation of intersection points in distorted linear segments. The threshold is set experimentally to 0.5.

##### Adding Tolerance Points

**Figure 8.**
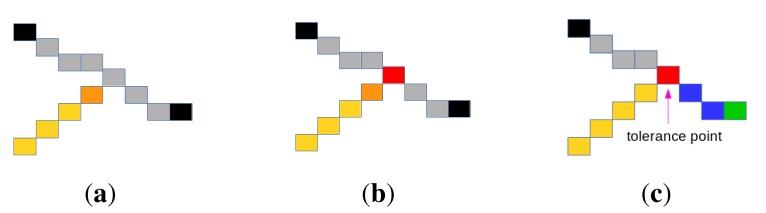
Illustration of adding a tolerance point.

The previous steps may lead to undesirable situations where two adjacent rides are represented by two segments that do not have a common point. This is due to the stop conditions of the segment extraction process (Step 2). This configuration is illustrated in Figure 8a, where the light brown and gray segments do not have a common point. The calculated extremity of the light brown segment is the brown point, while the desired extremity is the red point in Figure 8b. To deal with this shortcoming, the concept of “tolerance point”, described in Algorithm 3, is introduced. Added to optimize the creation of segments in order to meet the real shape of the roof, a tolerance point (if it exits) is an edge point that is already referenced in a segment. As shown in Figure 8c, adding the tolerance point allows obtaining a configuration in which the initial gray segment is subdivided into two segments (blue and gray ones). This transformation contributes to making the 2D roof model more faithful to the real shape of the roof. Adding a tolerance point is authorized only if the angle between the vector formed by the last five points of the first segment (light brown segment in Figure 8a) and the vector formed by its extremity (brown point in Figure 8a) and a candidate tolerance point belonging to the second segment (gray segment in Figure 8b) is less than π/2, *i.e*., when the scalar product between these vectors is greater than zero.


**Algorithm 2:** Intersection point calculation
**begin** seg ← segment candidate to subdivision vectorLength(point A; point B) ← length of the vector connecting the point A to the point B ListPoint ← all the points of the segment seg  (startPt,endPt) ← seg extremities globalLength ← vectorLength(startPt ; endPt) maxLength ← 0 tempLength ← 0 **for**
*each point pts of ListPoint*
**do**  **if**
*(pts!*= *startPt AND pts!*=*endPt)*
**then**   tempLength ← vectorLength(pts ; startPt) + vectorLength(pts ; endPt)   **if**
*(tempLength* > *maxLength)*
**then**    maxLength ← tempLength;    interPoint ← pts;  **if**
*((maxLength / globalLength) > threshold)*
**then**   **return** interPoint **else**  **return** NULL


**Figure 9.**
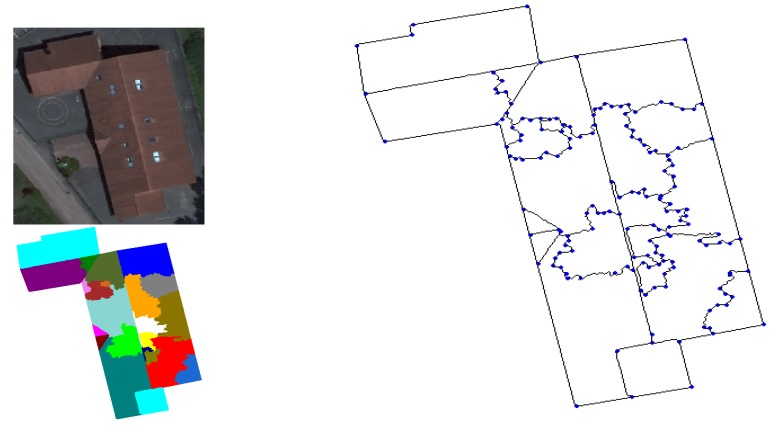
Example of 2D modeling (initial image, pre-segmented image using watershed-regions and the corresponding 2D model).

Figure 9 illustrates an example of the 2D modeling of roof ridges by segments. As detailed above, theses segments are obtained from the pre-segmented image (provided by watershed regions) on which the described four steps of the 2D modeling procedure are applied successively.


**Algorithm 3:** Adding tolerance points.
**begin** vectorLength(point A; point B) ← length of the vector connecting the point A to the point B PtEnd ← last point of the segment Pt5BeforeEnd ← 5th before PtEnd PtsCandidates ← retrieve all edge points around the last point PtEnd PtNext ← Choose the best point among PtsCandidates using Algorithm 1 scalarProduct ← scalar product [Pt5BeforeEnd ; PtEnd] with [PtEnd ; PtNext] **if**
*(scalarProduct* > *0)*
**then**  Add the point PtNext as a tolerance point


#### 4.1.2. Region Merging

In this section, we present our region merging strategy mainly based on the analysis of the region adjacency graph (RAG) of the pre-segmented image and a merging criterion adapted to orthophotoplan particularities using the 2D roof ridge modeling technique.

**Figure 10.**
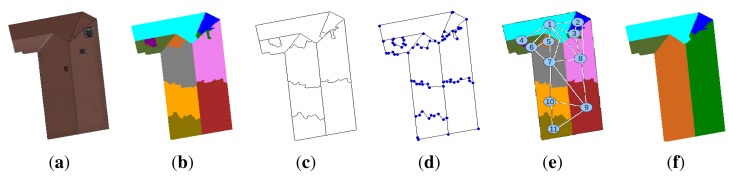
Region merging strategy. (**a**) Initial image; (**b**) pre-segmented image by the watershed algorithm; (**c**) edge extraction; (**d**) model of segments; (**e**) region adjacency graph (RAG) of the pre-segmented image; (**f**) final segmentation.

It is important to notice that, after the segmentation provided by the watershed algorithm, the straight line aspect of a boundary of two regions belonging to two main roof sections is always respected (for example, the roof sections labeled 7 and 8 in Figure 10e). In this case, the boundary is characterized by at most two segments. However, the straight line aspect is not respected when it concerns a boundary separating two regions resulting from an over-segmentation (for example, the roof sections labeled 8 and 9 in Figure 10e). In this case, the boundary is characterized by at least five segments.

Since a ridge separating the roof sections corresponds to a straight line segment, a simple merging criterion consists of merging the neighboring regions when the common boundary is not a straight line segment. For that, our merging strategy uses the number of segments *δ_seg_* modeling the boundary between two regions candidates to the fusion. Indeed, for two adjacent regions, we extract their common boundary and determine (by the 2D roof ridge modeling technique previously described) the number of segments *δ_seg_* composing it. If *δ_seg_* is greater than one, the two regions are merged. A drawback of this merging criterion is that it produces an under-segmentation of the image. This problem is generally due to the poor quality of the extracted ridges, which can be caused by the non-detection of a roof light or a chimney located on a boundary. Thus, the boundary is modelized by several segments. For example, in Figure 10, the non-detection of the window located between the regions, labeled 2 and 8, leads to a boundary modeled by three segments. To deal with this shortcoming, information for the boundaries and regions can be exploited: the mean difference *ϱ̅_R_i_,R_j__* (Equation (2)) of two regions *R_i_* and *R_j_* belonging to two different roof sections (for example, the regions labeled 7 and 8 in Figure 10e) should be lower than the contrast *ξ_R_i_,R_j__* (Equation (1)) observed on the common boundary. On the contrary, if we consider a boundary between two regions resulting from an over-segmentation and belonging to the same roof section (for example, the regions labeled 8 and 9 in Figure 10e), the propriety mentioned above is not respected. In the proposed merging strategy, radiometric (the mean difference of regions and the contrast on the boundary) and geometrical quantities (the straight line segment aspect) are used conjointly. This permits taking into account the orthophotoplan particularities and, thus, obtaining the best segmentation results. The region merging algorithm is given below (Algorithm 4).

- *Im* is a gradient image.
- *R_i_* and *R_j_* are two neighboring regions candidates to the fusion.
- *p*_1_ and *p*_2_ are two pixels of the image.
- *δ_seg_* is the number of segments modeling the common boundary between the regions *R_i_* and *R_j_*.
- *ξ_R_i_,R_j__* is the contrast on the boundary between the regions *R_i_* and *R_j_*.
  (1) ξRi,Rj=1η∑p1∈Rimaxp2∈Vp1,p2∈Rj(|Im(p1)−Im(p2)|)
  where *η* is the number of pixels of the common boundary between the regions *R_i_* and *R_j_* and *V_p_*_1_ is the neighborhood of *p*_1_.
- *ϱ̅_R_i_,R_j__* is the mean difference of the regions *R_i_* and *R_j_*.
  (2) ϱ¯Ri,Rj=|1η1∑p1∈RiIm(p1)−1η2∑p2∈RjIm(p2)|
  where *η*_1_ and *η*_2_ are, respectively, the number of pixels of the regions *R_i_* and *R_j_*.

**Algorithm 4:** Region merging algorithm.
*δ_seg_* ← Modeling(boundary(*R_i_,R_j_*));*ϱ̅_R_i_,R_j__* ← abs(Mean(*R_i_*)-Mean(*R_j_*));*ξ_R_i_,R_j__* ← Contrast(boundary(*R_i_,R_j_*));**if**
*δ_seg_* ≥ *5 OR (ξ_R_i_,R_j__* ≤ *ϱ̅_R_i_,R_j__*
*AND 3* ≤ *δ_seg_* ≤ *4)*
**then** the region with a minimum label absorbs the region with the maximum label, i.e. label =min(label(*R_i_*), label(*R_j_*)) **if**
*label*==*label(R_i_)*
**then**  ∀ p ∈ *R_j_*  p=label **else**  ∀ p ∈ *R_i_*  p=label


### 4.2. Segmentation Using Watershed Lines

**Figure 11.**
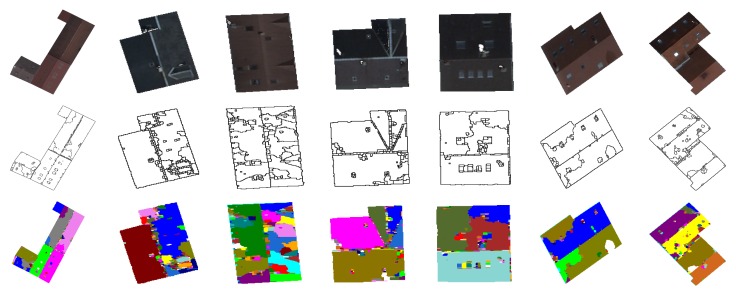
Segmentation results with watershed lines. (**Top**) Initial image, (**middle**) edges segmentation and (**bottom**) corresponding labeled image.

In order to calculate the image of edges, we have used an accurate algorithm introduced by Vincent and Soille [56,57] and belonging to the watershed algorithm by immersion. The gradient image is considered as an altitude map or a topographic relief. It is used to distinguish homogeneous and heterogeneous zones of the image. The relief is flooded by the minima of the gradient image. When two retention basins meet, a watershed line is created to separate them. Intuitively, for the application to image analysis, the watershed lines represents the location of pixels that best separate dark objects (regional minima), in terms of gray level difference (contrast). The advantage of watershed line segmentation is that it leads to closed and fine adjacent contours, including all edges of the image. Nevertheless, the major drawback of this method is the high sensitivity to noise and local irregularities in the gradient image, which implies generally an over-segmentation. Figure 11 illustrates some segmentation results obtained by the watershed line-based algorithm applied to the simplified images with the appropriate couple of colorimetric invariant/gradient. The algorithm produces a strong over-segmentation of the roofs, but the majority of roof objects (chimneys, roof light, roof sections, *etc*.) are detected.

## 5. Watershed Regions and Watershed Line Cooperation

**Figure 12.**
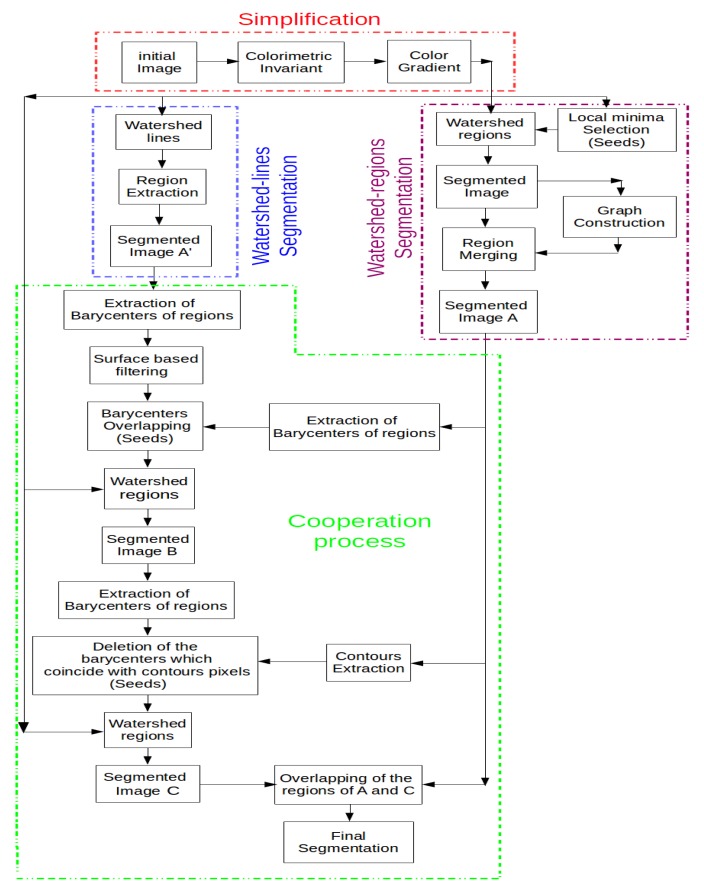
Synopsis of the watershed region and watershed line cooperation.

In the previous section, we presented the watershed algorithms (region and line based) and the region merging strategy. We propose integrating these methods into a single cooperative segmentation scheme to achieve satisfactory segmentation results. Indeed, as we will show, this region/edge cooperative process is able to exploit the advantage of each segmentation method. Watershed region segmentation coupled with the region merging strategy allows dealing with the over-segmentation problem in the orthophotoplans. However, it presents the drawback of losing chimneys, roof lights and some roof sections. These components are either lost in the region merging step or not detected by the watershed algorithm. The non-detection of these components can be justified by the lack of or the bad position of seeds. Watershed lines are used to overcome this drawback. Indeed, this allows detecting the majority of the roof components, but produces an important over-segmentation on the boundaries between the different roof sections. To improve the quality of orthophotoplan segmentation, the cooperation between the watershed algorithms and the region merging strategy is then suitable. Figure 12 illustrates, in three steps, the principle of the proposed cooperation process:

1. The first step consists of preparing the input image to be used by the watershed algorithms (regions and lines) by applying the best couple of invariant/gradient, as defined in Section 3.
2. The second step consists of segmenting the image by watershed regions and watershed lines. These two segmentation techniques are applied in an independent and parallel way. On the one hand, the image is segmented by watershed regions, and then, the region merging process is performed in order to limit the over-segmentation phenomena. At this stage, we obtain a segmented image called *A* in Figure 12. On the other hand, the image is segmented by watershed lines followed by region extraction (each closed edge permits obtaining a region). From the produced segmented image, called *A*' in Figure 12, the barycenter of each region is calculated. The barycenters corresponding to regions with a surface greater than a threshold experimentally fixed at 600 (pixels) are ignored. This allows keeping only barycenters of regions that correspond to real roof objects (roof lights, chimneys and roof sections) that are not detected by watershed regions.
3. The final step corresponds to the cooperation process. First, watershed regions are applied again on the image where the seeds used correspond to the overlapping of the barycenters of the regions of the segmented image *A* and those extracted and filtered (as explained above) from the regions of the segmented image *A*'. This first cooperation stage produces a segmented image (called B in Figure 12) containing the roof components, which are not detected when applying only watershed regions. After that, the edges of the segmented image *A* are exploited to remove small regions due to the fluctuations of intensity on the boundaries of the segmented image *B*. To do this, the barycenters of the regions of the segmented image *B* that coincide with an edge-pixel of the segmented image *A* are ignored. Then, watershed regions are applied again on the image using the obtained barycenters as seeds. This produces a segmented image called *C* in Figure 12. The image *C* should contain almost all structures of interest characterizing the roof. The final segmentation *F* is produced by overlapping all regions of the segmented image *A* and the regions of the segmented image *C* with a surface lower than a threshold experimentally fixed at 800 pixels, *i.e*.,
  (3) $$F=\left\{\overset{N}{\underset{i=1}{\cup }}\underset{\in A}{R_{i}}\right\}\cup \left\{\overset{M}{\underset{j=1}{\cup }}\underset{\in C}{R_{j}^{'}}/\eta \left(R_{j}^{'}\right)<\tau \right\}$$
  where N and M are, respectively, the numbers of regions of the segmented images *A* and *C, η*( $R_{j}^{'}$) is the number of pixels of the region $R_{j}^{'}$ and *τ* is a threshold set experimentally at 800.

## 6. Experimental Results

In this section, we present the experimental results of the different steps of the proposed approach, namely: (1) the choice of the optimal couple of colorimetric invariant/gradient in the simplification step; (2) the performance of the region merging strategy applied to watershed region segmentation; (3) the segmentation results of the watershed region and watershed line cooperation process; and (4) the comparison results with five popular segmentation techniques of the literature. We point out that the tests presented in this paper have been performed on orthophotoplans with a spatial resolution of 10,000 pixels by 10,000 pixels (1 pixel = 16 cm) provided by Communauté de l'Agglomération Belfortaine (CAB 2008). One hundred heterogeneous roofs were extracted from these orthophotoplans using their known ground track and were segmented and evaluated with the Vinet criterion [64]. To better understand and visualize the performance of each step of our proposed approach, we have chosen to show the results in both qualitative and quantitative ways.

### 6.1. Choice of the Best Couple of Invariant/Gradient

**Figure 13.**
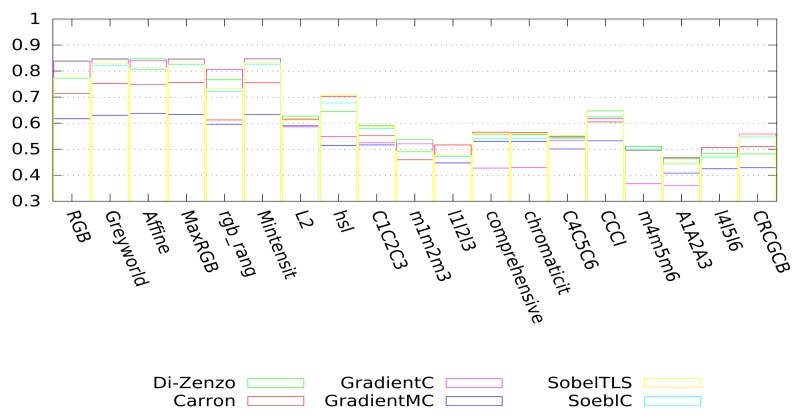
Quality of the segmentation on color images (using the VINETcriterion) according to the couple colorimetric invariant/color gradient.

**Figure 14.**
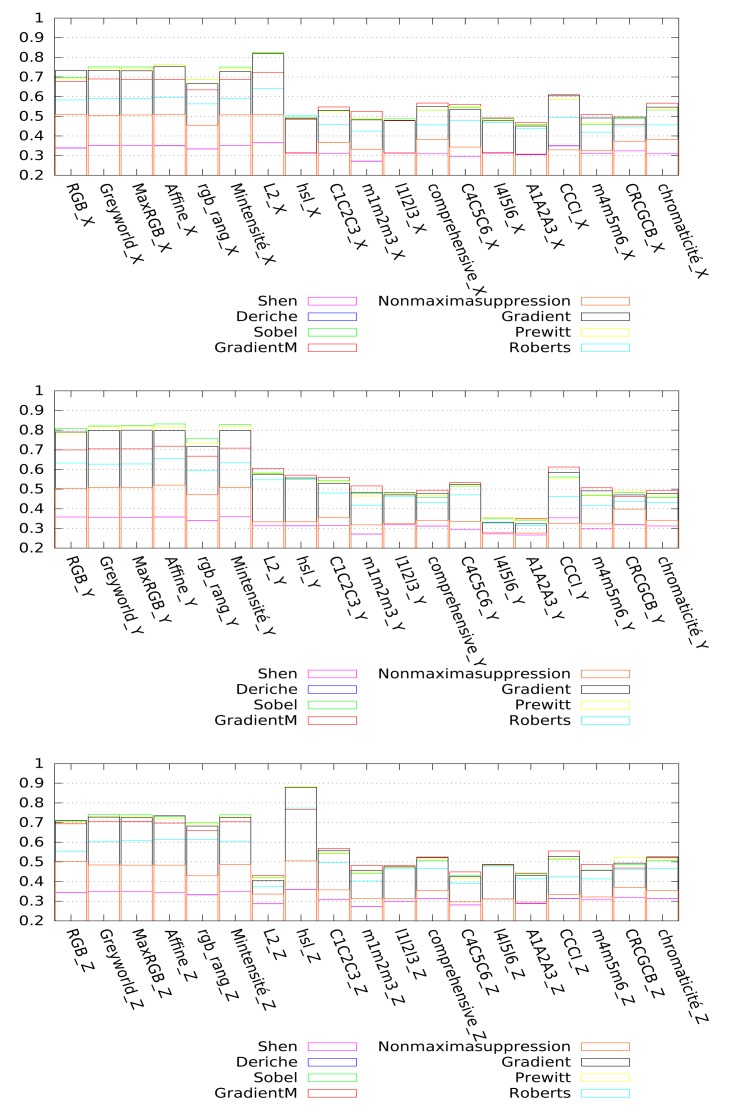
Quality of the segmentation on gray level images (using the Vinet criterion) according to the couple gray level invariant/gray level gradient. Segmentation results on the X component (**top**), Y component (**middle**) and Z component (**bottom**) of the color image.

As indicated in Section 3, we propose to simplify the initial image using an appropriate couple of colorimetric invariant/gradient. The tests presented below are separated into two categories. The first one corresponds to the evaluation of the couple colorimetric invariant/color gradient (*cf*. Figure 13), and the second one corresponds to the evaluation of the couple gray level invariant/gray level gradient (*cf*. Figure 14). The gray level invariants are obtained from the three components of the colorimetric invariants: X, Y and Z represents, respectively, the first, second and third components of a given colorimetric invariant. For example, hsl-Y corresponds to the second component, *i.e*., the saturation (S) of the hsl color space. We point out that for better readability, we have only presented 18 of the best invariants among the 24 tested. For both figures (Figures 13 and 14), each bar represents the mean value of Vinet calculated on all images of the test database according to the couple invariant/gradient tested, and the higher the value is, the better the results are.

Considering the color gradients (cf. Figure 13), greyworld, affine normalization, RGB-rang, maximum-intensity normalization and MaxRGB give very good segmentation results, whatever the color gradient used. Nevertheless, the couple greyworld/Di-Zenzo remains the best one. We can rank the best couples as follows: greyworld/Di-Zenzo, Mintensity/Di-Zenzo, MaxRGB/Di-Zenzo, affine normalization/Di-Zenzo and Mintensity/GradientC.

Considering the gray level gradients (*cf*. Figure 14), the segmentation results depends highly on the color component used. Indeed, for the three X, Y and Z components of the colorimetric invariants greyworld, affine normalization, RGB-rang, maximum-intensity normalization and MaxRGB, we obtain satisfying results (the values of Vinet are high compared to those obtained with the components of the other colorimetric invariants) whatever the gradient used. If we are interested only in the best component, one can notice that the X component of the L2 normalization (L2-X) and the Z component of the hsl space (hsl-Z) give the best segmentation results. Finally, we can rank the best couples as follows: HSL-Z/Prewitt, HSL-Z/Sobel, HSL-Z/GradientM, Mintensity-Y/Sobel and L2-X/Sobel.

Considering these tests, we opted to use the couple of greyworld/Di-Zenzo as the optimal couple of invariant/gradient for image simplification purposes.

### 6.2. Performance of the Region Merging Algorithm

**Figure 15.**
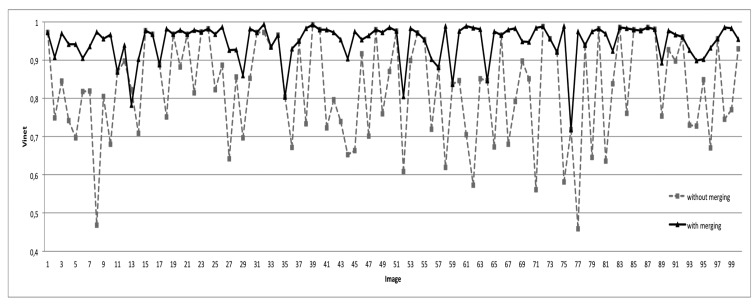
VINET-based evaluation of the segmentation results obtained without and with the proposed merging strategy.

**Figure 16.**
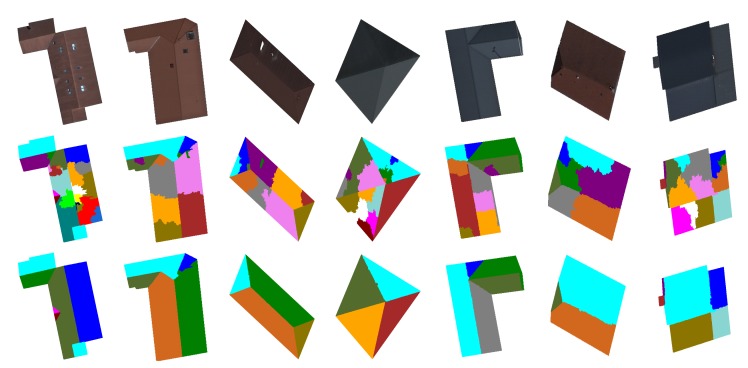
Segmentation results based on the proposed region merging strategy. Initial images (**first row**); segmented images without (**second row**) and with the proposed merging strategy (**third row**).

In order to study the ability of the merging criterion to deal with the over-segmentation problem, we present in Figure 15 the segmentation results generated without and with the proposed merging technique. One can see clearly that the proposed region merging strategy allows for considerably improving the segmentation results. Indeed, the Vinet value is higher (*i.e.*, the segmentation results are better) when the region merging procedure is associated with the segmentation step performed by watershed regions. The use of the region merging strategy allows increasing the rate of good segmentation from 83% (with watershed regions only) to 94% (with watershed regions + fusion). This is justified by the fact that the strategy was able to merge all non-significant regions due to over-segmentation, while preserving the roof sections. Figure 16 illustrates the segmentation results and highlights the gain provided by the region merging strategy. For example, if we consider the first image (first column of Figure 16), the rates of good segmentation obtained without/with fusion are 47% and 97%, respectively. The rate of 47% explains that the image is strongly over-segmented (the second image of the first column), whereas the rate of 97% shows that the image (the third image of the first column) agrees most closely with the corresponding ground truth. However, one can notice a loss of certain components of the roofs, such as chimneys, roof lights and some roof sections. We show in the next section how this loss can be compensated for through the proposed watershed region and watershed line cooperation process.

### 6.3. Performance of the Watershed Region and Watershed Line Cooperation Process

**Figure 17.**
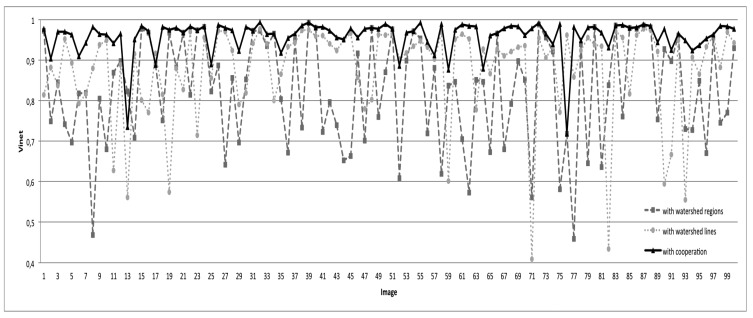
Evaluation results (with the Vinet criterion) according to the watershed regions, watershed lines and the cooperation process.

**Figure 18.**
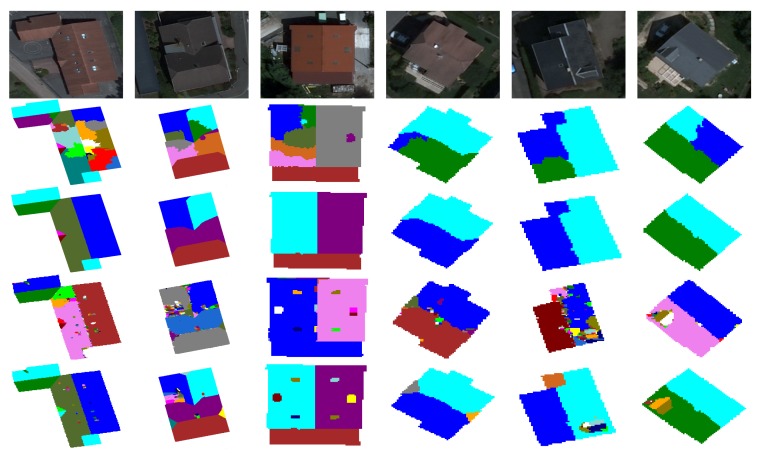
Segmentation results based on the proposed cooperation procedure. Initial images (**first row**); segmentation obtained by watershed regions (**second row**); segmentation obtained after the region merging step (**third row**); segmentation obtained by watershed lines (**fourth row**) and segmentation obtained with the proposed cooperation procedure (**fifth row**).

In this section, we present the segmentation results of the proposed overall approach. To highlight the importance of the different steps of our approach, we present in Figures 17 and 18 the segmentation results obtained by watershed regions only (the dashed line in Figure 17), by watershed-lines (the dotted line in Figure 17) and by the cooperation process (the black line in Figure 17). It appears from these results that the proposed cooperation approach allows for achieving the best segmentation results. Indeed, on average, the difference is important, since the rate increases from 83%, obtained by watershed regions, or 88%, obtained by watershed lines, to 96%, obtained by the watershed region/watershed line cooperation. We justify these results in Figure 18 by illustrating, on some images, the key steps of the proposed cooperation process. Note that the segmentation results obtained only with watershed-regions are not satisfactory, since the images are over-segmented (*cf*. Figure 18, Row 2). The region merging procedure allows for increasing the results, but several objects of the roofs, such as roof lights, chimneys, etc., are lost (cf. Figure 18, Row 3). The outcome obtained only by watershed lines allows keeping these objects, but produces an important over-/under-segmentation (*cf*. Figure 18, Row 4). As expected, the proposed cooperation procedure exploits the advantage of each method and then provides much better results. Indeed, the segmented images agree most closely with the corresponding ground truth, and most of the roof lights and chimneys are present (*cf*. Figure 18, Row 5).

**Figure 19.**
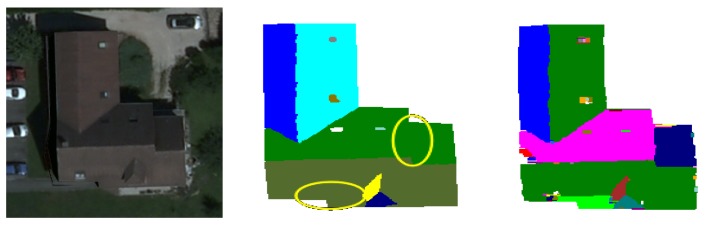
Illustration of an example for which the segmentation produced by our approach (the image of the second column) is less satisfactory than that obtained with the watershed-lines (the image of the third column).

We note that the segmentation obtained by the cooperation process is not as good as the segmentation obtained by watershed regions for one image of the test database (Image Number 12 in Figure 17). The same report is noted with watershed lines for four images of the test database (Image Numbers 16, 51, 63 and 75 in Figure 17). Figure 19 shows an example for which the quality of the segmentation achieved by the cooperation procedure is lower than that obtained with watershed lines used alone. One can notice that, contrary to watershed lines, the cooperation procedure did not succeed at detecting some objects from the roof (roof section, pane). These missing detections are probably due to the bad selection/placement of seeds during the watershed region or inappropriate merging operation. In general, these problems appear when faced with complex roofs composed of sections with juxtaposed or joined blocks that are not easily identifiable by humans.

### 6.4. Comparison with Other Methods

In this section, we study the ability of five popular segmentation techniques of the literature to segment the roof images and compare their performances with those obtained with our proposed segmentation approach. The segmentation algorithms that we have selected for the comparison are: mean shift-based segmentation algorithm (MS) [65], efficient graph-based segmentation algorithm (EGBIS) [66], statistical region merging (SRM) [67], JSEG unsupervised segmentation algorithm [33] and color structure code (CSC) [68]. These segmentation methods are well known and often used for image segmentation purposes. Most of these methods have several control parameters that are optimized for our application on all images of the test database. To obtain a meaningful comparison, each algorithm was tested over many possible combinations of input parameters. Table 1 shows the mean value of Vinet obtained on all of the images of the test database, with different possible combinations of input parameters for each segmentation method.

**Figure 20.**
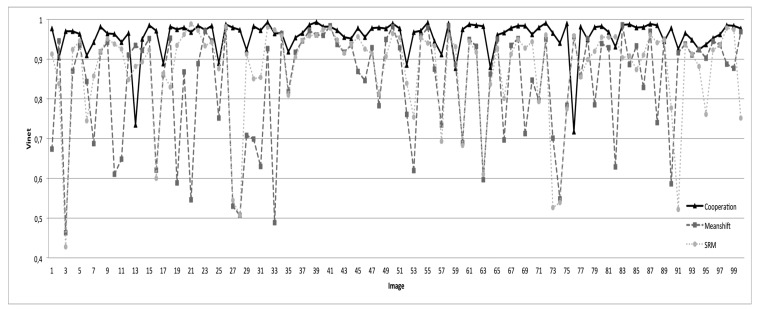
Evaluation results (with the Vinet criterion) according to our segmentation approach, mean shift and SRM.

Figure 20 illustrates the comparison results. For better readability, we have only presented the segmentation results obtained via SRM and mean shift methods, considered as better compared to the other tested state-of-the-art methods. The results presented here are obtained with the best parameters of each method. One can state clearly that the proposed segmentation approach behaves better than all of the tested methods. The rates of good segmentation are 96% with the proposed cooperation procedure *vs*. 87.5% with SRM *vs*. 84% with mean shift *vs*. 82% with CSC *vs*. 80% with EGBIS *vs*. 71% with JSEG.

**Figure 21.**
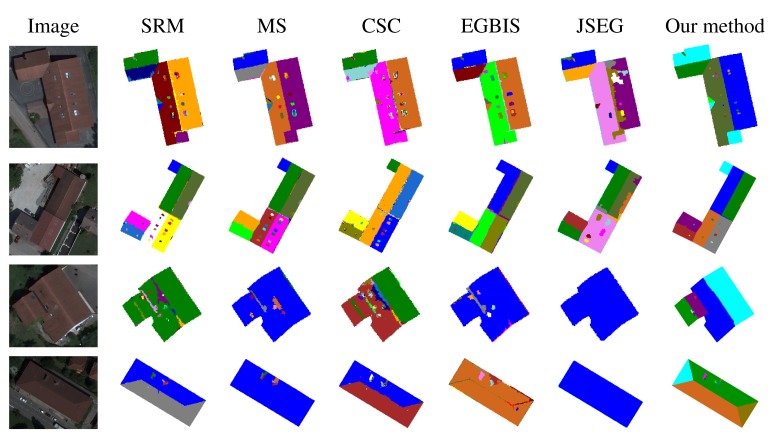
Segmentation results obtained with the five popular segmentation methods of the state of the art and with our method.

Figure 21 illustrates the segmented images produced by each algorithm for a set of images of the test database. Note that most of the tested algorithms give satisfactory results for some images, but fail and then become completely unsuitable for other images. This figure confirms the conclusion given from the analysis of Figure 20. Indeed, for all of the images in the two first rows of Figure 21, all of the tested methods give good segmentation results, where the produced segmented images agree most closely with the corresponding ground truth. One can note that for our method, most regions of interest of roofs (roof sections, roof lights, chimneys, *etc*.) are present. In contrast, the segmented images in the two last rows of Figure 21 have poor qualities where the images are extremely under-segmented. It is important to notice that our proposed method is more robust and fluctuates less compared to the five popular method of the state of the art. This leads us to conclude that these segmentation methods are not suitable for orthophotoplans when considering the requirements of our application. It is also useful to define our proposed segmentation method.

## 7. Conclusions

In this paper, we presented a robust hybrid roof segmentation method applied to aerial images (orthophotoplans). It is based on a cooperative process that consists of taking into account the advantages of watershed region-based coupled with a region merging strategy and watershed line-based segmentation techniques. As has been noticed, neither watershed regions nor watershed lines used alone gave accurate segmentation results considering the requirements of our application. Integrating their results in the same segmentation scheme contributes to a better segmentation. In watershed region-based segmentation, pre-processing and post-processing steps were used. The pre-processing step consists of simplifying the input image with an appropriate couple colorimetric invariant/gradient optimized for the application to limit artifacts and illumination changes. The post-processing consists of using an efficient region merging procedure based on a 2D roof ridge modeling technique in order to deal with the over-segmentation problem caused by the watershed region-based segmentation algorithm. The first segmentation technique leads to very promising segmentation of the roofs. It causes, however, a loss of chimneys, roof lights and some roof sections. In watershed line-based segmentation, we only used a pre-processing step by applying the optimal couple colorimetric invariant/gradient. Contrary to the watershed region-based segmentation technique, watershed line-based segmentation did not provide good results in general. However, it was able to detect all regions of interest of the roofs. That is why the idea of combining the advantages of each watershed-based segmentation method was useful. Judging by the results and their analysis, the cooperation process allowed for obtaining good segmentation with better detection of the objects of the roofs (roof lights, chimneys, *etc*.). Indeed, the segmentation results obtained by the proposed approach were very satisfying and closer to what was expected with reference images. Finally, the ability of five popular segmentation techniques of the literature to segment the roofs in our application was studied. Comparison of our method with these techniques showed that they did not satisfy the requirements of our application and their results were much lower than those obtained by the proposed method. Although the proposed approach has recorded very good performance, if the resolution or contrast decreases, it could be useful to revalue the best couple of invariant and gradient in the pre-processing step, adjusting the (alpha and beta) parameters of the seed reduction technique and the size of the small structures characterizing the roof.

In future works, we envisage considering multiple databases acquired in different conditions (resolution, contrast, noise, *etc*.) in order to optimize and determine the parameters of the proposed approach automatically. Furthermore, we plan to propose an automatic extraction of roofs from orthophotoplans instead of using their known ground tracks.

## Figures and Tables

**Table 1.** Mean value of the Vinet criterion obtained for five segmentation methods of the state-of-the-art (with several values for the control parameters) and for the proposed method. SRM, statistical region merging; CSC, color structure code; EGBIS, efficient graph-based segmentation; MS, mean shift.

**Methods**	**Parameters**	**Mean Value of Vinet**
SRM	Q = 600	85%
	Q = 800	85.6%
	Q = 1,500	87,5%
	Q = 3,000	86%
CSC	t = 5	67.5%
	t = 8	82%
	t = 12	81.5%
	t = 15	78.5%
EGBIS	*σ* = 0.3, *k* = 100	48.5%
	*σ* = 0.4, *k* = 200	80%
	*σ* = 0.4, *k* = 300	74%
	*σ* = 0.5, *k* = 200	80%
MS	*hs* = 3, *hr* = 6	59%
	*hs* = 3, *hr* = 8	84%
	*hs* = 8, *hr* = 2	77%
	*hs* = 10, *hr* = 5	73%
JSEG	*m =* 0.1, *q* = 10	70%
	*m* = 0.1, *q* = 100	67%
	*m* = 0.4, *q* = 80	71%
	*m* = 1, *q* = 100	67%
Our approach	*α* = 10, *β* = 15	96%
